# Cu-catalyzed trifluoromethylation of aryl iodides with trifluoromethylzinc reagent prepared in situ from trifluoromethyl iodide

**DOI:** 10.3762/bjoc.9.277

**Published:** 2013-11-08

**Authors:** Yuzo Nakamura, Motohiro Fujiu, Tatsuya Murase, Yoshimitsu Itoh, Hiroki Serizawa, Kohsuke Aikawa, Koichi Mikami

**Affiliations:** 1 Department of Applied Chemistry, Graduate School of Science and Engineering, Tokyo Institute of Technology, 2-12-1-H-113 O-okayama, Meguro-ku, Tokyo 152-8552, Japan

**Keywords:** organo-fluorine, Ruppert–Prakash reagent, trifluoromethyl, trifluoromethylation, trifluoromethyl zinc

## Abstract

The trifluoromethylation of aryl iodides catalyzed by copper(I) salt with trifluoromethylzinc reagent prepared in situ from trifluoromethyl iodide and Zn dust was accomplished. The catalytic reactions proceeded under mild reaction conditions, providing the corresponding aromatic trifluoromethylated products in moderate to high yields. The advantage of this method is that additives such as metal fluoride (MF), which are indispensable to activate silyl groups for transmetallation in the corresponding reactions catalyzed by copper salt by using the Ruppert–Prakash reagents (CF_3_SiR_3_), are not required.

## Introduction

Organo-fluorine compounds have received considerable attention in the fields of biomedical chemistry, agrochemistry, and organic material science due to their unique chemical, biological, and physical properties [1–6]. Particularly, trifluoromethylated compounds can be widely employed as one of the most effective analogues of bioactive compounds, because the trifluoromethyl group enhances the metabolic stability, lipophilicity, and bioavailability of these compounds [7]. As a result, trifluoromethylated compounds have been efficiently synthesized by both building-block methods which employ trifluoromethylated substrates and direct methods which employ trifluoromethyl reagents [7–9]. However, a nucleophilic trifluoromethylation by trifluoromethyl organometallic reagents such as lithium and magnesium, which are widely utilized in non-fluorine organic synthesis, cannot be used. These trifluoromethyl metal reagents are generally too unstable to prepare even at low temperature because of facile α-fluoro elimination generating the singlet difluoromethylene (:CF_2_) [10]. In contrast, trifluoromethylsilyl counterparts, so-called Ruppert–Prakash reagents (CF_3_SiR_3_), are highly stable, but reactive in the presence of fluoride, and hence the most versatile nucleophilic trifluoromethyl reagents [11–12]. The trifluoromethylzinc reagent (Zn(CF_3_)Br·2DMF), a stable solid, can also be used for the trifluoromethylation of aryl iodides, while stoichiometric amounts of copper(I) bromide are required to afford the more reactive trifluoromethyl copper (CuCF_3_) species by transmetallation [13–14]. However, these reagents are generally prepared from trifluoromethyl bromide (CF_3_Br), whose production is now prohibited because of the ozone depleting effect [15]. On the other hand, the trifluoromethylzinc reagent (Zn(CF_3_)I) formed in situ from trifluoromethyl iodide (CF_3_I) as an alternative trifluoromethyl source is utilized for trifluoromethylation reactions [10,16]. The preparation of the reagent followed by the reactions, however, requires ultrasonic irradiation and thus lacks reproducibility [16]. Therefore, the direct and reproducible preparation of the trifluoromethylzinc reagent and its application to trifluoromethylation reactions pose a particular challenge. Recently, Daugulis and co-workers reported the trifluoromethylation of aryl iodide catalyzed by copper(I) chloride with Zn(CF_3_)_2_ prepared in situ from TMP_2_Zn and fluoroform (CHF_3_), but only one substrate was investigated to provide the trifluoromethylated product only in a moderate yield [17]. As described above, much of the area of catalytic trifluoromethylations with trifluoromethylzinc reagent has not been explored yet, compared to the area of catalytic trifluoromethylations with the Ruppert–Prakash reagents [7,11–12,18–21]. Herein, we report the trifluoromethylations of aryl iodides catalyzed by copper(I) salt with trifluoromethylzinc reagent prepared in situ from CF_3_I and Zu dust. The trifluoromethylated aromatic products are privileged skeletal key compounds in pharmaceutical science as shown in Mefloquine (Lariam^®^), Fluoxetine (Prozac^®^), Leflunomide (Arava^®^), Celecoxib (Celebrex^®^), Bicalutamide (Casodex^®^), Aprepitant (Emend^®^), and Nilutamide (Nilandron^®^).

## Results and Discussion

The preparation of the trifluoromethylzinc reagent Zn(CF_3_)I was initially examined in the context of the in situ Cu-catalyzed trifluoromethylation of aryl iodide **1** under various conditions. The results of the reaction are summarized in Table 1. After Zn(CF_3_)I was prepared in situ by the treatment of CF_3_I (ca. 5 equiv) with Zn dust (2 equiv) [22] in various solvents at room temperature for 2 hours, the reactions were explored by adding a catalytic amount of copper(I) salt and 1,10-phenanthroline (phen) [18–21] followed by aryl iodide **1a**. In this catalytic system, solvents showed significant effects on the preparation and catalytic reactivity of Zn(CF_3_)I. No reaction was observed in less polar solvents such as toluene (Table 1, entry 1). Even in THF, CH_3_CN, DMSO, and NMP, the desired product **2a** was barely or not at all obtained (Table 1, entries 2–5). Formation of Zn(CF_3_)I and reaction in DMF were found to proceed only with 44% yield (Table 1, entry 6). Replacement of DMF with DMPU provided the trifluoromethylated product **2a** in 70% yield, along with the undesired pentafluoroethylated product **3a** in 9% yield (Table 1, entry 7).

**Table 1:** Copper-catalyzed trifluoromethylation of aryl iodide **1a**.

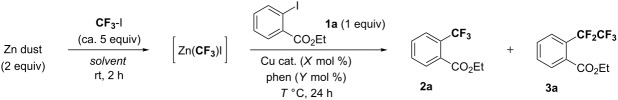					

entry	solvent	Cu cat.	(X, Y)	*T*	% yield (**2a**/**3a**)^a^

1	toluene	CuI	10, 20	50	n.r.
2	THF	CuI	10, 20	50	n.r.
3	CH_3_CN	CuI	10, 20	50	1/1
4	DMSO	CuI	10, 20	50	2/0
5	NMP	CuI	10, 20	50	n.r.
6	DMF	CuI	10, 20	50	44/8
7	DMPU	CuI	10, 20	50	70/9
8	DMPU	CuI	10, 10	50	93/7
9	DMPU	CuI	10, 10	rt	61/2
10	DMPU	CuI	10, 10	40	67/5
11	DMPU	CuI	10, 10	65	77/10
12	DMPU	CuCl	10, 10	50	92/8
13	DMPU	CuTC	10, 10	50	92/6
14	DMPU	CuI	2, 2	50	95/2

^a^Yields were determined by ^19^F NMR analysis by using benzotrifluoride as an internal standard.

The product **3a** should be derived from CuCF_2_CF_3_ generated by an insertion of difluoromethylene (:CF_2_) decomposed from CuCF_3_ into CuCF_3_ [23]. Decreasing the loading of phen, the yield of product **2a** was further increased to exceed the level of 90% yield (Table 1, entry 8). The reaction was promoted even under the milder reaction conditions at room temperature (61% yield), but the highest yield was obtained at 50 °C (Table 1, entry 8 vs entries 9–11). A further change from CuI to CuCl and CuTC led to comparable results (Table 1, entries 12 and 13). With only a 2 mol % loading of both CuI and phen ligand, the yield of **2a** was further increased to a higher level (95% yield) along with an increased selectivity with only 2% yield of **3a** (Table 1, entry 14).

The ligand effect was further investigated in DMPU at 50 °C and phen was preferable to other diamine ligands (Table 2, entries 1–3 vs entry 4). Surprisingly, even in the absence of phen, it was found that the reaction smoothly proceeded to give a comparably high yield of product **2a** (Table 2, entry 6). The reaction with a shorter reaction time of 2 hours indicated that the phen ligand slightly accelerated the reaction by the coordination to CuCF_3_ species, when compared to the reactions performed without the ligand (Table 2, entry 5 (78% yield) vs entry 6 (68% yield)). In the absence of CuI, no coupling product was obtained even in the presence of phen (Table 2, entries 7 and 8).

**Table 2:** Effect of ligands in copper-catalyzed trifluoromethylation.

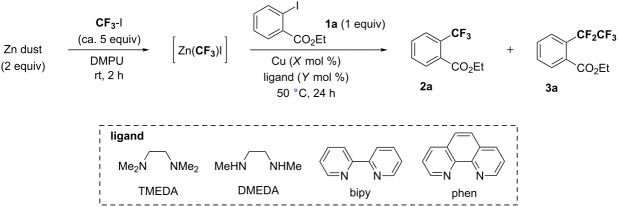			

entry	ligand	(X, Y)	% yield (**2a**/**3a**)^a^

1	TMEDA	10, 10	89/3
2	DMEDA	10, 10	83/3
3	bipy	10, 10	86/9
4	phen	10, 10	93/7
5	phen	2, 2	95/2 (78/1)^b^
6	–	2, 0	97/3 (68/1)^b^
7	–	0, 0	n.r.
8	phen	0, 2	n.r.

^a^Yields were determined by ^19^F NMR analysis by using benzotrifluoride as an internal standard. ^b^Values in parentheses are yields obtained with a reaction time of 2 hours.

**Figure 1:**
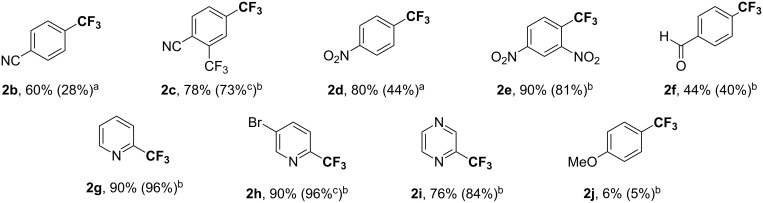
Copper-catalyzed trifluoromethylation of various aryl iodides. Yields were determined by ^19^F NMR analysis by using benzotrifluoride as an internal standard. Values in parentheses are yields obtained under the reaction conditions without phen. Conditions: CF_3_I (2.5 × X equiv) and Zn dust (X equiv) in DMPU, then CuI (Y mol %), phen (Y mol %) and **1** (1 equiv) at 50 °C for *t* hours. ^a^X = 4, Y = 2, *t* = 48. ^b^X = 2, Y = 10, *t* = 24. ^c^Isolated yields: **2c**, 70%; **2h**, 90%.

With the reaction conditions established in DMPU at 50 °C in the presence of a catalytic amount of CuI and phen, the scope and limitation of this method were evaluated. The results are shown in Figure 1. The use of the electron-deficient aryl iodides **1b–f** bearing nitrile, nitro, formyl, and trifluoromethyl groups led to the corresponding products **2b–f** in moderate to high yields. The reactions of heteroaryl iodides **1g–i** were also catalyzed to provide the corresponding products **2g–i** in good to excellent yields. In the case of **1h**, the formation of a CF_3_ group occurred only at the position of iodide, and bromide remained intact during the course of reaction. It was found that an increase of the yield in the presence of the phen ligand depends on the particular substrate, while the yields were within the same range except for **2b** and **2d**. Unfortunately, aryl iodide **1j** bearing the electron-donating methoxy substituent extremely decreased the reactivity, even when increasing the catalytic amounts of CuI and phen.

**Scheme 1:**
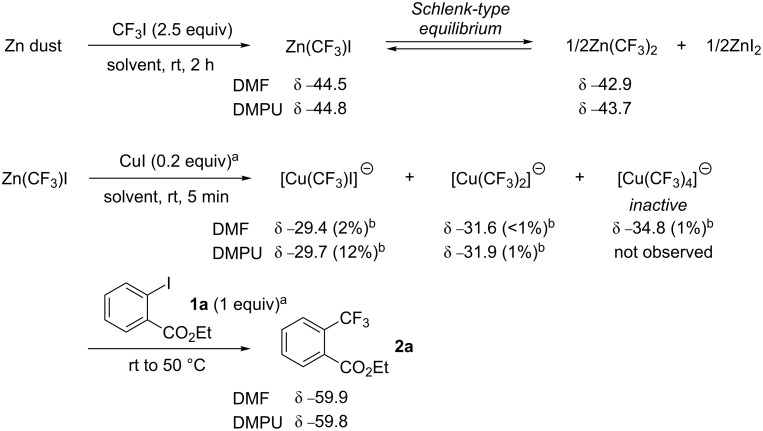
Observation of CuCF_3_ species in ^19^F NMR spectrum. ^a^Equivalents based on Zn(CF_3_)I. ^b^Yields based on CuI.

In order to gain an insight into each step of the catalytic trifluoromethylation with a trifluoromethylzinc reagent, a ^19^F NMR analysis in DMF and DMPU was performed (Scheme 1). At the initial stage, Zn(CF_3_)I which readily causes a Schlenk equilibrium with Zn(CF_3_)_2_ and ZnI_2_ [14,24] was prepared in situ from CF_3_I and Zn dust in 60–80% yields in both solvents. The addition of CuI (0.2 equiv) to a DMPU solution of Zn(CF_3_)I led to the transmetallation of the CF_3_ group from zinc to copper even at room temperature. Two singlet peaks of the cuprate species, [Cu(CF_3_)I]^−^ (−29.7 ppm) and [Cu(CF_3_)_2_]^−^ (−31.9 ppm) were observed in 12% and 1% yields, respectively [23,25–26]. By replacing DMPU with DMF as a solvent, the transmetallation was found to be less efficient than in DMPU. Moreover, the inactive copper species [Cu(CF_3_)_4_] ^−^ (−34.8 ppm) [27] was obtained. The neutral CuCF_3_ species (−26.3 ppm), which formed by the direct cupration of fluoroform in DMF [28] and was active even with aryl iodides bearing electron-donating substituents such as **1j** [28–30], was not observed in both solvents. Thus, the addition of aryl iodide **1a** led to the formation of the trifluoromethyl coupling product **2a** (−59.8 ppm) even in the absence of phen, involving the consumption of the cuprates [Cu(CF_3_)I] ^−^ and [Cu(CF_3_)_2_] ^−^. The use of DMF as a solvent led to a gradual increase of the peak assigned as inactive [Cu(CF_3_)_4_] ^−^ during the course of the reaction.

**Scheme 2:**
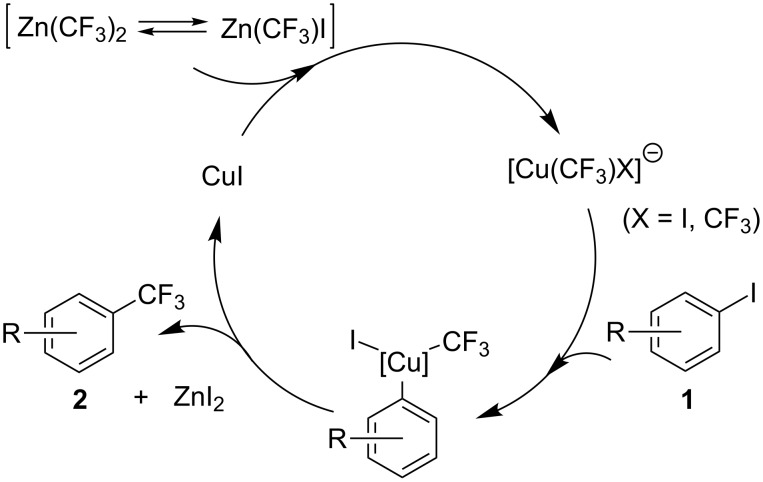
Proposed mechanism of copper-catalyzed trifluoromethylation.

The mechanism of the coupling reaction can thus be visualized by the following catalytic cycle (Scheme 2). At the first step, the transmetallation of the CF_3_ group to CuI from Zn(CF_3_)I or Zn(CF_3_)_2_ affords the active cuprate species, [Cu(CF_3_)X]^−^ (X = I, CF_3_). Subsequently, the oxidative addition to the cuprate of aryl iodide **1**, and the reductive elimination gives the desired cross-coupling product **2** together with the formation of ZnI_2_.

## Conclusion

In summary, we succeeded in the aromatic trifluoromethylation catalyzed by copper(I) salt with a trifluoromethylzinc reagent prepared in situ from trifluoromethyl iodide and Zn dust in DMPU. The catalytic reaction proceeded to provide moderate to high yields and a high selectivity of the trifluoromethylated product under mild reaction conditions. The advantage of this catalytic reaction is that additives such as metal fluoride (MF), which are indispensable to activate silyl substituents for the transmetallation in the corresponding catalytic reactions by using the Ruppert–Prakash reagents, are not necessary. Additionally, with some substrates, the reaction conditions without a ligand led to higher yields than reaction conditions with a ligand such as 1,10-phenanthroline. Further studies on highly efficient trifluoromethylation and difluoromethylation reactions with trifluoromethylzinc reagents are under way.

## Experimental

### Typical procedure for copper-catalyzed trifluoromethylation of aryl iodide

To the suspension of zinc powder (without activation, 65.4 mg, 1.0 mmol, Aldrich 99.995% purity) in DMPU (0.5 mL), trifluoromethyl iodide (ca. 2.5 mmol, sufficiently dissolved in the solution) was added at room temperature under argon atmosphere. After the solution was stirred for 2 h at room temperature, CuI (1.9 mg, 0.01 mmol, 2 mol %), 1.10-phenanthroline (1.8 mg, 0.01 mmol, 2 mol %), and then aryl iodide **1a** (138.0 mg, 0.5 mmol) were added. The reaction mixture was stirred at 50 °C for 24 h. After cooling to room temperature, the yield of product **2a** was determined by ^19^F NMR analysis by using benzotrifluoride (BTF) as an internal standard. Except for **2c**, all trifluoromethylated products **2** exhibited the same ^1^H, ^13^C, and ^19^F NMR spectra as reported before [14,17,29,31–36].

### 2,4-Bis(trifluoromethyl)benzonitrile (**2c**)

To the suspension of zinc powder (without activation, 65.4 mg, 1.0 mmol, Aldrich 99.995 % purity) in DMPU (0.5 mL), trifluoromethyl iodide (ca. 2.5 mmol, sufficiently dissolved in the solution) was added at room temperature under argon atmosphere. The solution was stirred for 2 h, and CuI (9.5 mg, 0.05 mmol, 10 mol %) and 4-iodo-2-(trifluoromethyl)benzonitrile (**1c**, 148.5 mg, 0.5 mmol) were added. The reaction mixture was stirred at 50 °C for 24 h. The reaction mixture was quenched with H_2_O (5 mL), and then Et_2_O (5 mL) was added. After filtration over celite, the organic layer was separated, and the aqueous layer was extracted with Et_2_O (5 mL × 3). The combined organic layer was washed with brine (10 mL), dried over Na_2_SO_4_, and evaporated. The resulting crude product was purified by silica gel column chromatography (pentane/Et_2_O 9:1) to give the product **2c** (83 mg, 70% yield) as a colorless liquid. ^1^H NMR (300 MHz, CDCl_3_) δ 8.06 (s, 1H), 8.03 (d, *J* = 8.2 Hz, 1H), 7.98 (d, *J* = 8.2 Hz, 1H); ^13^C NMR (400 MHz, CDCl_3_) δ 135.5, 135.0 (q, *J*_CF_ = 34.4 Hz), 134.0 (q, *J*_CF_ = 33.5 Hz), 129.3 (q, *J*_CF_ = 3.6 Hz), 124.1–123.9 (m), 122.3 (q, *J*_CF_ = 271.9 Hz), 121.6 (q, *J*_CF_ = 272.7 Hz), 114.1, 113.9; ^19^F NMR (282 MHz, CDCl_3_) δ −62.2 (s, 3F), −63.6 (s, 3F); HRMS–ESITOF (*m*/*z*): [M − H]^−^ calcd for C_9_H_2_F_6_N, 238.0091; found, 238.0086; FTIR (neat, cm^−1^) 2238, 1344, 1146, 1279, 1082.

### Observation of trifluoromethylcopper species in ^19^F NMR spectrum

To the suspension of zinc powder (without activation, 32.7 mg, 0.5 mmol, Aldrich 99.995% purity) in DMF or DMPU (0.75 mL), trifluoromethyl iodide (ca. 1.25 mmol, 2.5 equiv, sufficiently dissolved to the solution) was added at room temperature under argon atmosphere. After the solution was stirred for 2 h at room temperature, the remaining trifluoromethyl iodide was removed by bubbling argon through the solution for 15 min. To the solution was added CuI (19.0 mg, 0.1 mmol) at room temperature. After the reaction mixture was stirred for 5 min, the generation of cuprate species was monitored by ^19^F NMR analysis by using benzotrifluoride (10 μL, 0.0814 mmol) as an internal standard and sealed capillary filled with benzene-*d*_6_ for signal lock under argon atmosphere at room temperature.
